# Restoration of euthyroidism with levothyroxine: implications of etiology of hypothyroidism and the degree of residual endogenous thyroid function

**DOI:** 10.3389/fendo.2022.934003

**Published:** 2022-07-22

**Authors:** Jacqueline Jonklaas

**Affiliations:** Division of Endocrinology, Georgetown University, Washington, DC, United States

**Keywords:** quality of life, patient satisfaction, etiology, biomarkers, residual thyroid function, thyroid hormones

## Abstract

There are many thyroid-related factors that combine with non-thyroid-related factors in order to affect the patient response to treatment of hypothyroidism, in terms of their satisfaction with therapy. Some of the thyroid-derived factors include the etiology of the hypothyroidism and the amount of residual thyroid function that the patient retains. These two factors may be intertwined and affected by a third influence, the presence of thyroid peroxidase antibodies. The downstream consequences of the interactions between these three factors may influence both free thyroxine and free triiodothyronine levels, TSH concentrations, and various thyroid biomarkers. Evidence of the widespread importance of thyroid hormones can be inferred from the multiple genes that are regulated, with their regulation affecting multiple serum biomarkers. Thyroid biomarkers may extend from various well-known serum markers such as lipids and sex hormone-binding globulin to serum levels of thyroid hormone metabolites. Moreover, the interplay between thyroid hormones and biomarkers and their relative ratios may be different depending on the hypothyroidism etiology and degree of residual thyroid function. The ultimate significance of these relationships and their effect on determining patient-reported outcomes, quality of life, and patient satisfaction is, as yet, poorly understood. However, identification of better biomarkers of thyroid function would advance the field. These biomarkers could be studied and correlated with patient-reported outcomes in future prospective studies comparing the impact of various thyroid hormone therapies.

## Introduction

Thyroid hormone is the mainstay of therapy for patients with hypothyroidism. The correlation between biochemical parameters, clinical parameters, and satisfaction with thyroid hormone therapy has been a subject of spirited discussion for some time. This commentary reflects upon how biochemical parameters respond to thyroid hormone therapy and presents some of the evidence regarding the accompanying response of the clinical parameters, while considering etiology of hypothyroidism and residual thyroid function. Some thoughts are also entertained as to the significance of the biochemical and clinical response and their complex interface with patient quality of life (QOL) and satisfaction with therapy.

## Restoration of the euthyroid state

### Biochemical euthyroidism

Following the development of primary hypothyroidism, thyroid function is typically restored by administration of levothyroxine (LT4) (1, 2). Based on consideration of the interactions of the hypothalamic-pituitary-thyroid axis hormones, biochemical euthyroidism is generally defined as achievement of a serum thyroid stimulating hormone (TSH) within the normal laboratory reference interval (1). Sometimes the TSH goal may be modified to achieve a narrower range of values (1), for example, values that approximate the mean TSH of 1.4 ± 0.02 mIU/L that was documented in a reference population free of disease or other influences upon thyroid parameters (3). However, although LT4 therapy, with any needed adjustments for its absorption and metabolism, can be relied upon to normalize TSH, the normalization of endogenous thyroid hormones cannot be replicated. Total thyroxine (T4) or free thyroxine (FT4) are higher than the native state and total triiodothyronine (T3) or free triiodothyronine (FT3) may be lower (4–6). TSH suppression can, however, contribute to normalizing the FT3 (5). In addition, ratios of FT4 to T3 are higher in the treated state (4), while FT3 to FT4 ratios are lower (5, 6) (see **Table 1**).

**Table 1:** Comparison of thyroid hormone levels in native euthyroidism versus treated post-surgical hypothyroidism.

Author	Jonklaas (Ref 4)		Ito (Ref 5)		Gullo (Ref 6)	
Thyroid state	Before thyroid-ectomy	After thyroid-ectomy, taking LT4	Before thyroid-ectomy	After thyroid-ectomy, taking LT4	Before thyroid-ectomy	After thyroid-ectomy, taking LT4
Number of patients	50	50	135	135	3,875	1,811
Mean or median TSH mIU/L	1.18 ± 0.58	1.30 ± 1.89	1.65 (0.99-2.48)	0.21 (0.04- 1.02)	1.4 (0.90- 2.10)	1.2 (0.69 - 2.20)
Mean or median FT4 ng/dL or *pmol/L*	1.05 ± 0.19	1.41 ± 0.29	1.01 ± 0.11	1.39 ± 0.18	*13.8* *(12.0 – 15.4)*	*15.4* *(14.2 - -17.6)*
Mean T3 ng/dL (IA)	129.3 ± 26.7	127 ± 27.9	–	–	–	–
Median FT3 pg/ml or *pmol/L*	–	–	3.01 (2.87-3.19)	2.92 (2.71 – 3.19)	*4.47* *(3.85 - 4.94)*	3.70 (3.73 – 4.31)
Mean ratio (FT4 x100)/T3	0.85 ± 0.22 ⇔	1.15 ± 0.29 ⇑	–	–	–	–
Mean or median ratio FT3/FT4 (Italics indicate ratio obtained using FT4 and FT3 in pmol/L)	–	–	3.01 ± 0.35 ⇔	2.17 ± 0.31 ⇓	*0.32* *(0.27 – 0.37)* ⇔	*0.24* *(0.20- 0.28)* ⇓

Mean ± SD or Median (interquartile range) ⇑ = higher ⇓ = lower ⇔ = same.

### Clinical euthyroidism

**Figure 1:**
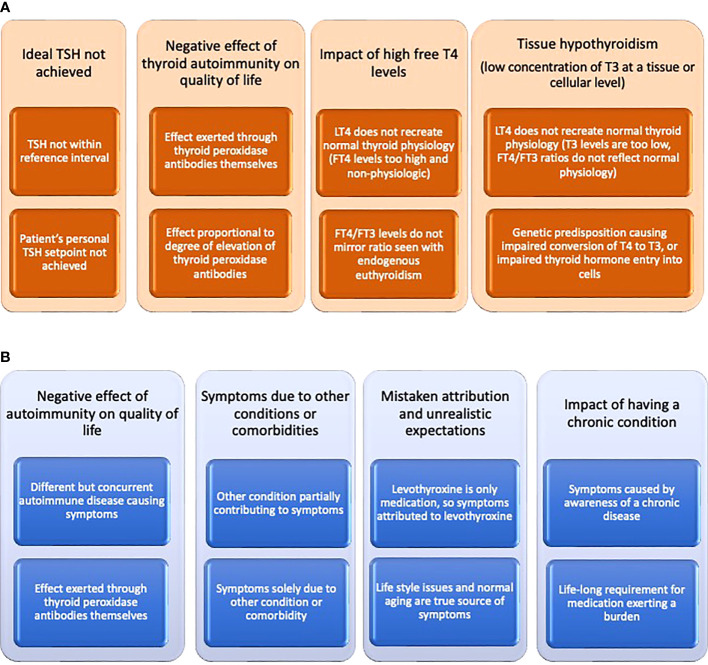
**(A)** Potential explanations offered for impaired quality of life despite treatment of hypothyroidism that may be thyroid-related. **(B)** Potential explanations offered for impaired quality of life despite treatment of hypothyroidism that may be thyroid-unrelated.

Despite the biochemical serum signature of endogenous euthyroidism differing from the treated euthyroid state, most patients with hypothyroidism report resolution of their symptoms with LT4 therapy (1, 2). However, a variable proportion of patients, typically reported as approximately 15%, continue to have persistent symptoms (7–10). These symptoms can include fatigue, low energy levels, weight management difficulties, low mood, impaired memory, and brain fog (11, 12). Many potential explanations for these unresolved symptoms can be offered, and these can be broadly divided into thyroid-related and thyroid-unrelated causes, although there may be overlap between these categories (2) (see **Figures 1A, B**). In all likelihood more than one of these explanations may be in play in a particular patient. One of the potentially most compelling explanations is based on deficient T3 concentrations at a serum, tissue, or cellular level. A recent review has addressed the complex relationship between serum and tissue levels of T3 in various tissues and the deiodinases contributing to T3 production and clearance (13).

Attempting to address the reduced serum T3 levels in LT4-treated patients, trials of combination therapy with both LT4 and liothyronine (LT3) have overall not achieved greater success in ameliorating symptoms of dissatisfaction, reduced QOL, or impaired memory and cognitive function than monotherapy with LT4 (1, 14, 15). However, these trials have many shortcomings, including use of a short acting LT3 preparation, failure to recruit patients who are the most symptomatic, and therefore potentially more likely to benefit from therapy, insufficient use of patient-reported outcomes, and being of short duration and statistically underpowered. Anecdotal data (11, 16) and a *post-hoc* analysis of a recent trial (17) hint at benefits from animal thyroid products providing combination therapy and either synthetic or natural combination therapy respectively. Given these issues, the potential explanation that unresolved symptoms may stem from low T3 levels at a tissue or cellular level has not been excluded (13, 14, 18). Biomarkers of euthyroidism that could reveal this tissue hypothyroidism have been investigated in animal studies (19, 20), but have not been studied in a rigorous manner in humans and deserve further attention (21, 22). Further study of thyroid-responsive genes or thyroid hormone metabolites could potentially shed light on markers of thyroid hormone action that may hold promise to more accurately delineate thyroid status in the future (23–26).

## Common etiologies of primary hypothyroidism

There are several common causes of primary hypothyroidism which all involve varying degrees of destruction of thyroid tissue (see **Table 2**). Hashimoto’s thyroiditis is probably most notably characterized by varying degrees of residual thyroid function (see next section) ranging from the mild decrement in thyroid function associated with subclinical hypothyroidism to the complete absence of thyroid function that occurs following the culmination of complete autoimmune destruction of the thyroid gland. At the other end of the spectrum is the hypothyroidism that precipitously occurs following total thyroidectomy in which there is absence of any endogenous thyroid function, and following which a patient becomes severely hypothyroid in about 4 weeks without LT4 replacement (27–29). Intermediate degrees of hypothyroidism may result from radioactive iodine ablation for treatment of Graves’ disease and external beam radiation for treatment of head and neck malignancies, with the degree of hypothyroidism being affected by the radiation dose utilized and the time elapsed since the treatment was applied. The etiology and severity of the hypothyroidism are thus closely linked and will in turn impact the LT4 dose that is required to normalize the serum TSH (30).

**Table 2:** Potential differences between primary hypothyroidism of various etiologies.

Etiology	TSH	Presence of TPO antibodies*	Serum T3	Serum free T4
**Prior to treatment**				
Subclinical Hashimoto’s hypothyroidism	Elevation of varying degrees	Yes	Normal	Normal
Subclinical (other etiologies)	Elevation of varying degrees	Usually no	Normal	Normal
Overt Hashimoto’s hypothyroidism	Substantial Elevation	Yes	Reduced or normal	Reduced
Radioactive iodine (RAI) ablation for hyperthyroidism	Elevation of varying degrees (RAI dose and time dependent)	Usually no	Reduced or normal	Reduced
External beam radiation for non-thyroid malignancy	Elevation of varying degrees (radiation dose and time dependent)	Usually no	Reduced or normal	Reduced
Hemithyroidectomy	Elevation of varying degrees	Usually no	Reduced or normal	Reduced
Total thyroidectomy	Substantial Elevation	Usually no	Reduced	Reduced
**Following treatment**				
Hashimoto’s thyroiditis	Normal	Yes, potentially declining over time	Normal or low-normal	Normal, high normal, or elevated
Radioactive iodine ablation for hyperthyroidism	Normal	Usually no	Normal, low-normal, or low	High normal or elevated
External beam radiation for non-thyroid malignancy	Normal	Usually no	Normal, low-normal, or low	High normal or elevated
Hemithyroidectomy	Normal	Usually no	Normal, low-normal	Normal, high normal, or elevated
Total thyroidectomy	Normal	Usually no	Low-normal or low	High normal or elevated

*TPO antibodies = thyroid peroxidase antibodies.

## Influence of etiology of hypothyroidism upon response to therapy

With the potential to redress hypothyroidism of any of these etiologies with supplementation with LT4 to normalize the serum TSH (see **Table 2**), the question is whether the biochemical parameters are fully restored and symptoms of hypothyroidism are equally addressed regardless of etiology.

### Biochemical response

As previously mentioned, thyroid parameters differ in athyreotic thyroidectomized patients treated with LT4, compared with those with native euthyroidism (4–6). In addition to documenting the lower T3 or FT3 levels and higher FT4 levels in thyroidectomized patients (4–6), an additional study has also examined other biomarkers of thyroid function in LT4-treated athyreotic patients (31). This study examined these biomarkers, such as sex hormone-binding globulin, bone alkaline phosphatase, low-density lipoprotein cholesterol, and acid phosphatase-5b levels, before and after thyroidectomy in patients with papillary thyroid cancer (31). The investigators found that mild TSH suppression was required to restore FT3 levels and also the concentrations of these biomarkers to their baseline pre-operative levels (31). These results could suggest that normal FT3 levels help restore other biomarkers of euthyroidism.

Interrelationships between TSH, FT4, and FT3 were examined in a study comparing three groups of individuals. These included control euthyroid individuals, LT4-treated patients, and LT4-treated patients with postsurgical hypothyroidism (32). The investigators proposed that FT3 concentrations were stable over the range of TSH values in untreated patients but were “unbalanced” in athyreotic LT4-treated patients. The authors documented thyroid volume in the three groups with results as follows: 16 mL in the untreated patients, 3 mL in the LT4-treated patients, and, 1 mL in the thyroidectomized LT4-treated patients. They postulated that patients with a thyroid volume of <5 mL had significantly reduced deiodinase activity, lower FT3 levels, and less capacity to increase their deiodinase activity with stimulation from a raised TSH level (32). Such hypotheses could have implications for inherent ability of athyreotic patients to maintain normal FT3 to FT4 ratios, other than through elevated FT4 levels, with attendant TSH lowering.

In summary, it is possible that normal biochemical parameters may be easier to achieve when the etiology of the hypothyroidism is associated with retention of some residual thyroid tissue.

### Clinical response

#### Significance of TSH

In patients with overt hypothyroidism, presumed to be Hashimoto’s hypothyroidism in etiology, initiated on LT4 therapy and monitored for one year, the target TSH achieved did not appear to influence other biomarkers of thyroid function (33). The TSH at one year was 1.06 ± 0.6 mIU/L in one group compared with 3.32 ± 0.7 mIU/L in a second group, and had been maintained relatively constant throughout the 12-month period. Serum lipid profile, body mass index, body composition, and bone mineral density did not differ between the two groups (33). The sole parameter that differed between the two groups was resting energy expenditure, which was higher in the group with the lower TSH level. During a second study, also examining the effect of different TSH targets in patients with primary hypothyroidism (presumed Hashimoto’s), patients were crossed over between three LT4 doses, with 8 weeks of therapy on each dose (34). The achieved mean TSH values were 0.3 ± 0.1 mIU/L, 1.0 ± 0.2 mIU/L, and 2.8 ± 0.4 mIU/L. None of the outcome measures, which included well-being, hypothyroid symptoms, QOL (General Health Questionnaire 28, Short Form 36, and Thyroid Symptom Questionnaire), cognitive function tests, and treatment preference, were different between the two groups (34).

A third study randomized patients with presumed Hashimoto’s hypothyroidism to either a maintained, higher, or lower dose of LT4 and measured outcomes at 6 months (35). QOL (36-Item Short Form Health Survey, Underactive Thyroid-Dependent Quality of Life Questionnaire), mood (Profile of Mood States, Affective Lability Scale), and cognition (executive function, memory) were all measured and after adjustment for multiple comparisons there were no differences between the groups despite the significant different between the achieved TSH values (35). The TSH levels were 1.85 ± 0.25, 3.93 ± 0.38, and 9.49 ± 0.80 mIU/L in the three groups. In the same patients the different TSH targets were also not associated with differences in energy expenditure, body mass, or body composition (36). Taken together (33–36), these results do not suggest that titrating LT4 therapy to different target TSH values within the normal reference interval has a significant impact on outcomes.

Based on the evidence from these three studies the concept that titrating serum TSH levels to a specific target within the normal range might be beneficial should be carefully considered when treating patients with hypothyroidism. These studies do not support this concept (33–36). Considerable energy is expended by physicians and patients in order to target specific TSH goals, when perhaps other adjustments in therapy may be more fruitful.

#### Significance of triiodothyronine levels

7The athyreotic state following total thyroidectomy is characterized by almost complete absence of endogenous T3 production due to lack of thyroid tissue or minimal remnant tissue. On the other hand, Hashimoto’s hypothyroidism, at least in its earlier stages, may still be associated with some endogenous T4 and T3 production. Thus, treatment of Hashimoto’s hypothyroidism may require less LT4 to normalize the TSH, and not be associated with as high FT4/T3 ratios as might be seen with athyreosis. Hypothyroidism caused by radioactive iodine ablation or external beam radiation may also, like the case of Hashimoto’s hypothyroidism, be associated with varying degrees of T4 and T3 deficiencies. If maintenance of serum T3 levels was required for symptom resolution, it might be anticipated that LT4 might be more successful for symptom relief for partially developed Hashimoto’s hypothyroidism than for postsurgical hypothyroidism. The same might be postulated for hypothyroidism that develops after radiation or radioactive iodine administration. An additional consideration is that based on animal studies normal serum FT3 levels do not necessarily provide normal T3 content in the brain (18, 19). This could be relevant to the discussion of symptoms in humans receiving therapy for hypothyroidism.

In a retrospective study TSH, FT4, and FT3 interactions and hypothyroid and hyperthyroid complaints were documented in athyreotic patients receiving LT4 replacement (37). Increasing LT4 dose had a more powerful effect on lowering the TSH than raising FT3 concentrations, leaving approximately 30% of the group with FT3 concentrations still in the lower half of the reference range despite a suppressed TSH. The authors also found that symptoms were related to all three thyroid parameters (TSH, FT4, and FT3). Relief of hypothyroid symptoms was most predicted by a combination of a low TSH and a FT3 level in the upper half of the reference range (37).

If higher serum T3 levels are associated with better resolution of hypothyroid symptoms, satisfaction with therapy may be more successfully achieved when the etiology of the hypothyroidism allows for normalization of FT3 levels.

#### Significance of thyroid peroxidase antibodies

Another factor that might also affect QOL in individuals treated for hypothyroidism is the presence of thyroid peroxidase (TPO) antibodies. TPO antibodies could potentially either cause, or be associated with, symptoms in those who have developed thyroid insufficiency and also in those who still maintain normal thyroid function. For example, comparing euthyroid individuals with TPO antibody titers greater than or less than 121 IU/mL revealed significantly greater rates of chronic fatigue (66% compared with 49%), lack of concentration (32% compared with 19%), and nervousness (68% compared with 39%) in those with the higher compared with the lower TPO antibody titers (38). A cross-sectional study also found that TPO antibody levels greater than 100 IU/mL significantly increased the risk of depression (odds ratio 3.0, 95% confidence interval 1.3–6.8) (39). A non-randomized study of thyroidectomy for benign thyroid disease in a group of individuals of whom only 25% were taking LT4 pre-thyroidectomy examined hypothyroid symptoms before and after thyroidectomy. There was no improvement in QOL in the group overall, although those with higher TPO antibody levels were more likely to have an improvement in emotional role and bodily pain components of the SF-36 (40).

An open label randomized trial published in 2019 examined the impact of thyroidectomy on patients with substantial elevation of their TPO antibodies who were biochemically euthyroid while taking LT4, but still had persistent symptoms (41). The primary outcome of the study was the general health score on the Short Form-36 Health Survey (SF-36). For those who underwent thyroidectomy there was a significant improvement in the general health score at 6, 12, and 18 months after surgery, as well as improvement in several subscores, and improvement in the score on a fatigue questionnaire. Those enrolled in the study had baseline TPO antibody titers greater than 2000 IU/mL, which decreased substantially in the surgery group by 6 months after surgery (41). Intriguing though the results of this study are, there are some cautions. Firstly, it is not known if such benefits in terms of symptom relief would also be seen in individuals who have lesser degrees of TPO antibody elevation or who have less marked symptoms. The baseline SF-36 general health score of study participants was 38, compared with a normative score of 75 in the general Norwegian population (42). In addition, there was, understandably, no placebo surgery and the duration of the response beyond 18 months is not known. In addition, the surgical risks in patients with Hashimoto’s disease need to be considered (43).

In summary, it seems likely that high titers of TPO antibodies are associated with residual symptoms in patients with hypothyroidism. However, this may not be actionable unless a means of reducing the level of the TPO antibodies, other than through such a permanent measure as thyroidectomy, is identified. Also, there is a risk that absence of residual thyroid tissue following thyroidectomy may contribute to dissatisfaction if the contribution of the residual tissue to maintaining T3 or FT3 levels is important.

## Degrees of residual thyroid function

**Figure 2:**
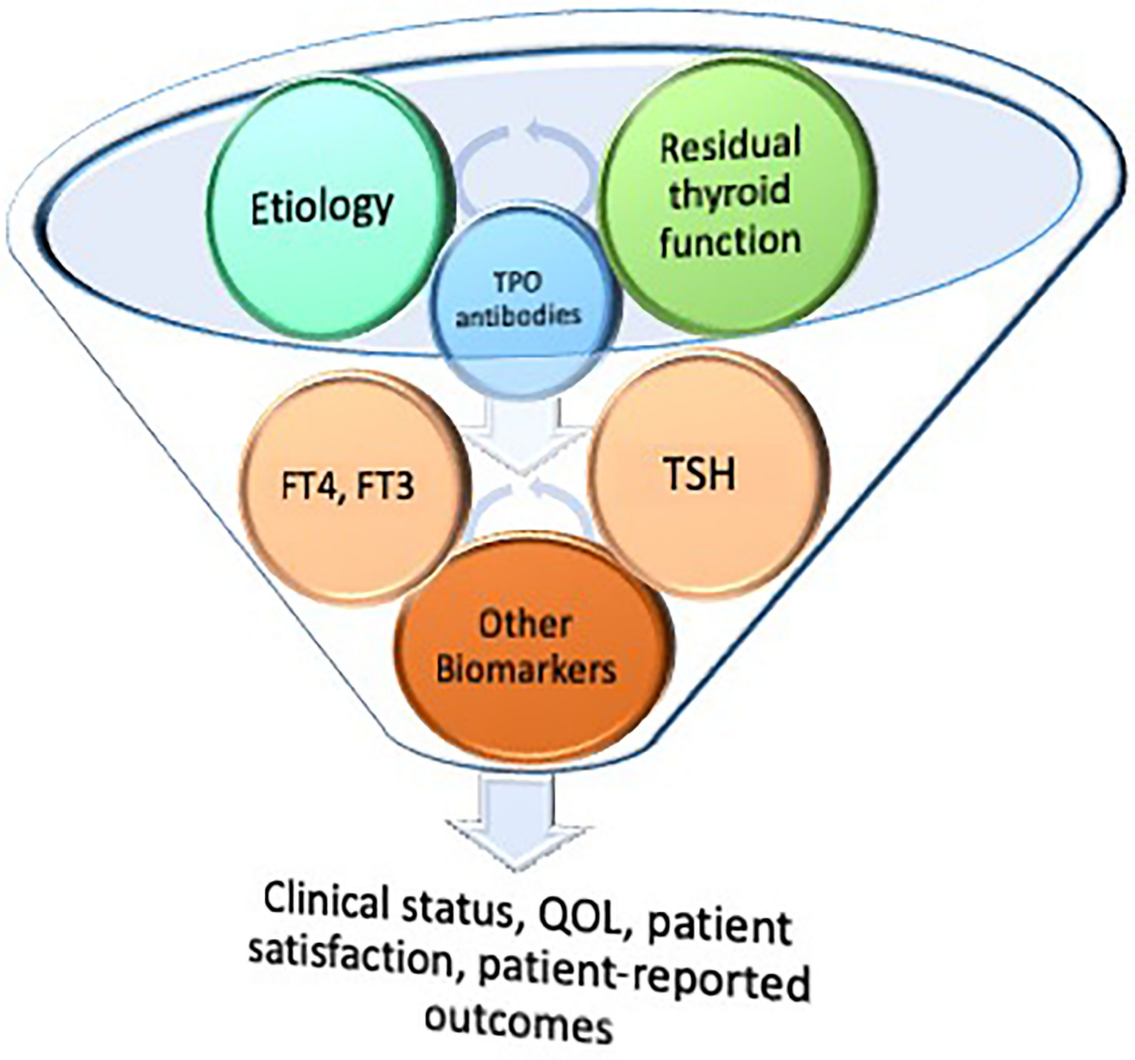
Hypothetical interaction between residual thyroid function and etiology of hypothyroidism.

The degree of residual thyroid function in a patient with hypothyroidism can run the gamut from subtle impairment to complete absence of native thyroid function. The former might be seen in Hashimoto’s thyroiditis, external beam radiation and radioactive iodine induced damage, and hemithyroidectomy. The latter can be seen in advanced Hashimoto’s hypothyroidism and after total thyroidectomy. Thus, it can be imagined that etiology of hypothyroidism and degree of residual thyroid function are closely linked and interrelated (**Figure 2**). Considering whether patients who retain some residual thyroid function may be more likely to obtain satisfactory therapy and symptom resolution may be complicated by other factors, such as the presence of TPO antibodies, radiation-induced side effects, and the possibility of surgical complications.

**Figure 3:**
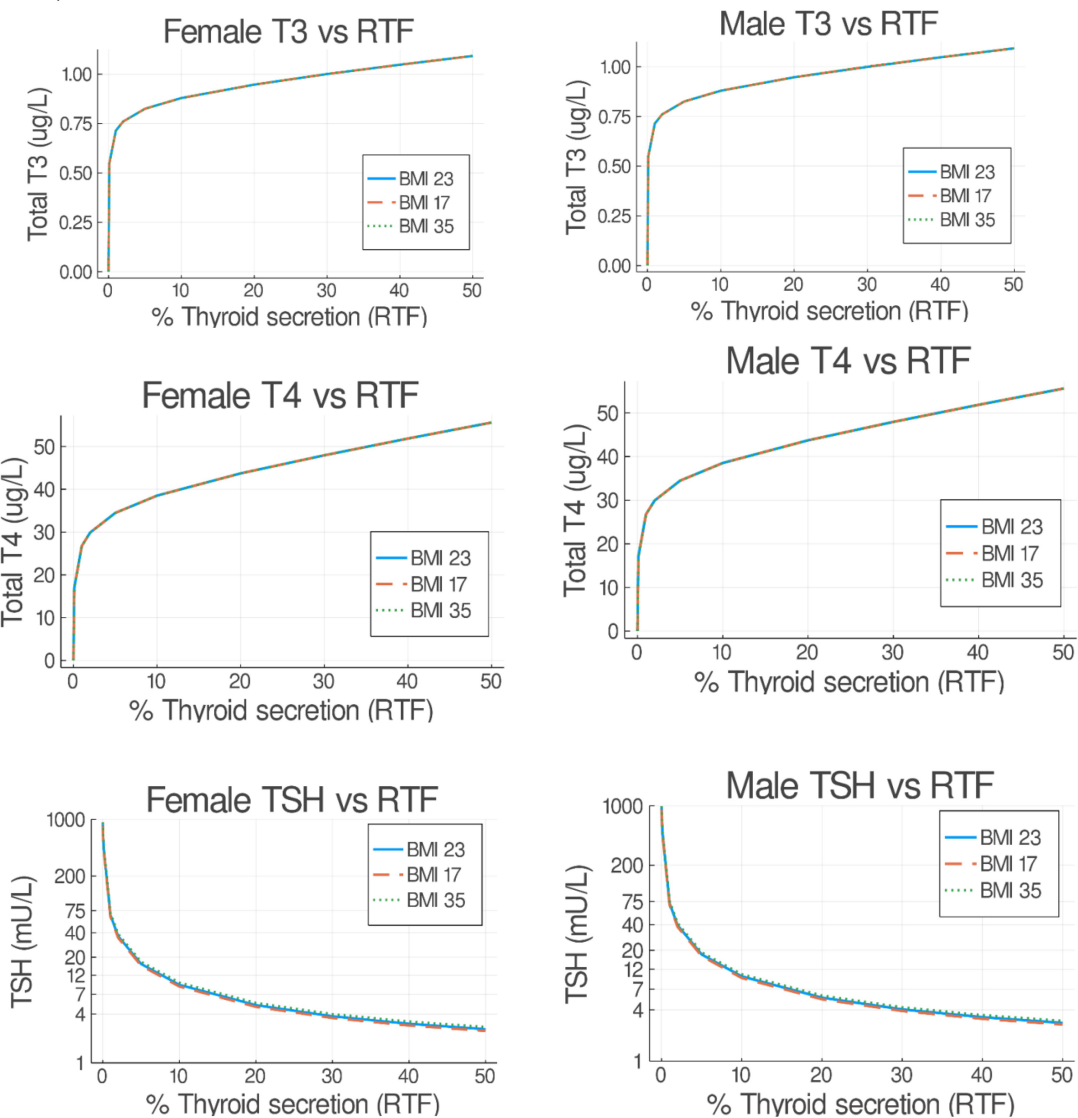
Predicted steady state serum T3, T4, and TSH concentrations versus residual thyroid function (RTF) values. Untreated obese, normal and underweight patients were simulated for 50 days. The final steady state T3, T4, and TSH simulation values are shown on the abscissa for each RTF value on the ordinate axis, separated into female (left hand side) and male (right hand side) plots. (From Cruz-Loya et al., Ref 45).

Mathematical modeling can be conducted to demonstrate the decline in thyroid hormones and the rise in TSH that occur as thyroid function declines (44, 45). **Figure 3** shows the predicted steady state concentrations of TSH, T4, and T3 after 50 days a with a particular degree of residual thyroid functioning. The T4 and T3 concentrations in this model drop precipitously after approximately 20% of residual thyroid function is reached. As would be anticipated with the upregulation of the type 2 deiodinase activity seen with hypothyroidism (46, 47), T3 concentrations are better maintained than T4 concentrations as TSH concentrations rise.

## Influence of residual thyroid function upon response to therapy

### Biochemical response

A 2015 study examined the TSH, FT4 and FT3 concentrations in patients after they had undergone surgery for papillary thyroid cancer (48). The biochemical response to thyroid hormone replacement with LT4 after thyroid surgery differed according to whether the patient had undergone total thyroidectomy versus hemithyroidectomy. Furthermore, the biochemical parameters differed from individuals who were either started or not started on LT4 after lobectomy. The FT3 to FT4 ratio was highest in those patients who underwent lobectomy without LT4 replacement, was significantly lower in those who received LT4 replacement after lobectomy, and was lowest in those who had undergone a total thyroidectomy followed by LT4 replacement. A lower TSH and higher FT4 accompanied the lower FT3/FT4 ratio. Thus, the patients with a lobectomy alone had the highest FT3 levels, the highest TSH values (mean value 2.41 mIU/L), and the highest FT3/FT4 ratios (48). These data suggest that remnant thyroid tissue plays an important role in maintaining serum FT3 levels after surgery. This same study did not examine patient satisfaction with therapy. The same authors also conducted a study in patients who had undergone radioactive iodine therapy for hyperthyroidism with subsequent atrophy of their thyroid glands (49). They documented the residual thyroid volume in these patients and correlated it with the serum FT3 levels. When thyroid volume was either less than 5 mL or between 5 and 10 mL, patients had lower FT3 levels than control individuals with normal endogenous thyroid function. However, a residual thyroid volume of greater than 10 mL was associated with a FT3 level that did not differ from control individuals.

Both these studies (48, 49) illustrate the role of remnant thyroid tissue in contributing to the maintenance of serum FT3 levels.

### Potential clinical response

The response to initiating LT4 therapy for hypothyroidism may be impacted both by the relative degree of decline in the T4 and T3, as well as other factors specific to the etiology of the hypothyroidism. These factors might affect the thyroid hormone levels achieved (biochemical response) and potentially also the clinical response to therapy in terms of satisfaction and symptom relief. As mentioned previously, with mild Hashimoto’s hypothyroidism, T3 levels may be relatively protected and initiation of LT4 may not decrease the FT3/FT4 ratio as much as more advanced Hashimoto’s hypothyroidism. If a more normal FT3/FT4 ratio is associated with better response to therapy, fully advanced Hashimoto’s hypothyroidism may be more resistant to satisfactory treatment. In these cases of autoimmune hypothyroidism, there also may be other concomitant autoimmune diseases that also independently negatively impact QOL.

As a parallel to the example of mild versus severe Hashimoto’s hypothyroidism, it could be postulated that hypothyroidism following hemithyroidectomy may respond more satisfactorily with better resolution of symptoms than hypothyroidism following total thyroidectomy. This hypothesis is based on the assumption that the more protected T3 levels seen with a hemithyroidectomy allow for FT3/FT4 ratios that more closely approximate endogenous FT3/FT4 ratios and are also linked with better QOL and a greater degree of symptom resolution.

Patients contemplating thyroid surgery often express a wish to avoid total thyroidectomy (50). This may be for multiple reasons including the desire to retain some endogenous thyroid function and avoid LT4 replacement.

In one prospective study of patients who had undergone total thyroidectomy for papillary thyroid cancer, the authors documented several symptoms associated with thyroid function both before and after thyroidectomy (51). These symptoms included sweating, temperature tolerance, activity, bowel movements, appetite, skin wetness or dryness, hand and foot temperature, and hand tremor. The investigators documented better normalization of all these measures with a mild degree of TSH suppression that was also associated with a FT3 level that mirrored the pre-thyroidectomy FT3 concentration (51). Weight gain despite treatment of hypothyroidism is a major concern of many patients being treated for hypothyroidism. A retrospective study of weight trends in individuals with hypothyroidism treated to achieve a normal TSH showed more weight gain over a year period in patients with post-surgical hypothyroidism, compared with those with presumed autoimmune hypothyroidism (52). Both hypothyroid groups gained more weight than euthyroid controls. The weight gain was somewhat mitigated in a separate group of individuals being treated with TSH suppression therapy. The cause of the weight gain was not studied but could be postulated to be associated with parameters that were not fully normalized by the LT4 therapy, unless TSH suppression was achieved.

In a recent modelling study, it was possible to predict the specific doses of LT4 and LT3, given as combination therapy, that would be required to achieve mid-normal concentrations of T4 and T3 concentrations in a 70 kg individual. In addition, modeling of the published trials of combination therapy suggested that those trials that achieved higher simulated T3 levels were also the trials that reported either improvements in QOL, mood, and neurocognitive benefits or patient preference (44).

In parallel to the discussion of etiology of hypothyroidism, if higher serum T3 levels are associated with better resolution of hypothyroid symptoms, satisfaction with therapy may be more successfully achieved when the degree of residual thyroid function allows for normalization of FT3 levels. The etiology and the hypothyroidism and the degree of residual thyroid function are closely linked and probably cannot be studied separately.

## Conclusion

The downstream consequences of the interactions between etiology of hypothyroidism and residual thyroid function may influence both FT4 and FT3, TSH concentrations, and various thyroid biomarkers (see **Figure 2**). Thyroid biomarkers such as lipid profile, bone markers, and sex-hormone binding globulin may be relatively insensitive as biomarkers. However, biomarkers may extend to thyroid hormone metabolites, microRNAs, and thyroid-regulated genes that can be measured in peripheral blood cells (23–26). Moreover, the interplay between thyroid hormones and biomarkers and their relative ratios may be different depending on the hypothyroidism etiology and degree of residual thyroid function. Increasing the complexity of the situation is the potential influence of TPO antibodies and the need for a better understanding of the significance of retaining residual thyroid tissue. The ultimate significance of these relationships and their effect on determining patient-reported outcomes, QOL, and patient satisfaction are as yet poorly understood. Despite extensive data documenting FT4, FT3, and TSH during thyroid hormone therapy, the relative importance of these hormones and their ideal ratios is poorly understood. However, identification of better biomarkers of thyroid function that are accessible and could be combined into routine clinical practice would advance the care of patients with hypothyroidism. These biomarkers could be studied and correlated with patient-reported outcomes in future prospective studies comparing the long-term impact of various thyroid hormone therapies, while also paying attention to the relative risks and benefits of the therapies.

## Author Contributions

JJ is the sole contributor who conceived the idea and wrote the manuscript.

## Funding

JJ is supported by grant funding from the National Institutes of Health (UL1TR001409 and R01DE025822).

## Conflict of Interest

The author declares that the research was conducted in the absence of any commercial or financial relationships that could be construed as a potential conflict of interest.

The reviewer ACB is currently organizing a Research Topic with the author.

## Publisher’s Note

All claims expressed in this article are solely those of the authors and do not necessarily represent those of their affiliated organizations, or those of the publisher, the editors and the reviewers. Any product that may be evaluated in this article, or claim that may be made by its manufacturer, is not guaranteed or endorsed by the publisher.
